# Restrictive migration policies and their impact on HIV prevention, care and treatment services

**DOI:** 10.1186/s12961-024-01172-0

**Published:** 2024-08-05

**Authors:** Olabode Ekerin, Deborah Oluwaseun Shomuyiwa, Don Eliseo Lucero-Prisno, Oluwafemi Oluwaseun Agboola, Ayelawa Samuel Damilola, Silvia Ojonoka Onoja, Chisom Favour Chikwendu, Emery Manirambona

**Affiliations:** 1 School of Public Health, University of Port Harcourt, Port Harcourt, Nigeria; 2 The WiseUp Initiative for Good Health and Community Development, Kaduna, Nigeria; 3 Faculty of Pharmacy, University of Lagos, Lagos, Nigeria; 4 Department of Global Health and Development, London School of Hygiene and Tropical Medicine, London, United Kingdom; 5 State AIDS, Hepatitis and STI control Program, Department of Public Health, Ministry of Health, Osogbo, Osun State Nigeria; 6 Ibrahim Babangida Badamosi Specialist Hospital, Minna, Nigeria; 7 Dora Akunyili College of Pharmacy, Igbinedion University Okada, Okada, Edo State Nigeria; 8 College of Medicine and Health Sciences, University of Rwanda, Kigali, Rwanda; 9 Global Health Focus, Bujumbura, Burundi

**Keywords:** Migration, Policies, HIV, Country, Immigrant

## Abstract

Migration policies have a significant impact on population health, particularly for individuals living with human immunodeficiency virus (HIV). These policies not only determine who is allowed to enter a country but also influence which immigrants can access services provided by the government. Some countries continue to impose restrictions on HIV-positive individuals, justifying these measures as necessary to protect public health and mitigate healthcare and economic concerns. However, these restrictions lack a valid public health rationale. Due to social, economic and political constraints, restrictive migration laws hinder access to HIV prevention, care and treatment services for immigrants living with HIV. Immigrants face numerous challenges in accessing medication, adhering to treatment regimens and benefitting from HIV preventive efforts. This situation increases the risk of HIV infection and adverse health outcomes due to limited access to preventive programmes, social stigma and engagement in risky behaviours. Additionally, these restrictive migration rules negatively affect immigrants’ mental health. To improve the health of both immigrants and host communities, inclusive and evidence-based migration policies that address healthcare through public health and human rights lenses are required.

## Background

Migration policies play a crucial role in upholding state sovereignty by meticulously determining eligibility criteria for entries and departures. [1]. These policies extend beyond matters of immigration control and also encompass the important task of identifying eligible recipients of various services, ensuring equitable distribution of resources and promoting efficiency. However, they may exert a significant impact on the health of populations, particularly among immigrants living with human immunodeficiency virus (HIV) [2].

Migration policies significantly influence healthcare services delivery and their accessibility among immigrants living with HIV. Unfortunately, certain countries continue to impose restrictions on individuals who are HIV positive, prohibiting their entry or ongoing stay on the basis of threats of international spread of the virus [3]. These restrictions are justified by governments as measures to safeguard public health and prevent perceived burdens on healthcare and economic resources stemming from the presence of HIV-positive non-citizens. However, it is important to note that these justifications lack valid public health rationale [3], thus hampering health outcomes.

Due to a paucity of data, it is difficult to determine the exact extent to which migration affects the overall burden of HIV/acquired immunodeficiency syndrome (AIDS) globally. However, strong evidence indicates that migrants from countries with widespread HIV epidemics, notably those from sub-Saharan Africa, are disproportionately impacted by HIV compared with the local populations of host countries [4]. Conversely, the proportion of HIV/AIDS infections among migrants is generally lower than in their countries of origin, which can be explained by the “healthy immigrant effect” [4].

Moreover, disparities in healthcare access and structural discrimination contribute to the significant gap in the number of migrants living with HIV who can access treatment, despite global Antiretroviral therapy (ART) coverage reaching 76% [5]. In Western Europe, the proportion of migrants among those infected with HIV ranged from 20% to 40% [4]. HIV prevalence among migrant women aged 25–29 in KwaZulu-Natal, South Africa, has been shown to be as high as 63% [6]. Additionally, in South-East Asia, HIV prevalence among migrants to Thailand from neighbouring countries is up to four times higher than in the overall population [6].

Exploring the implications of those migration policies that restrict access to HIV prevention, care and treatment services, particularly for immigrants living with HIV, is essential to inform health policymakers and for advocacy. This article aims to provide a snapshot into these policies and their potential impact on the health and wellbeing of affected individuals in order to serve as a groundwork for further research on the need for more inclusive and evidence-based approaches to migration policies that prioritize both public health and human rights considerations.

## Current situation of restrictive migration policies and HIV services

Historically, numerous countries have implemented stringent immigration regulations specifically targeting individuals who are HIV positive [1]. These policies were initially rooted in misunderstandings and fears surrounding HIV transmission. Joint United Nations Programme on HIV/AIDS (UNAIDS) has played a pivotal role in advocating for the elimination of travel restrictions related to HIV, asserting that such restrictions are discriminatory and lack a scientific basis and providing guidance to countries on developing inclusive migration policies [3]. As global perspectives on individuals living with HIV have evolved and medical knowledge and treatments have advanced, some countries have recognized the discriminatory nature of these policies, thereby lifting or easing these restrictions [3].

While 203 jurisdictions worldwide, which includes countries, territories and areas, have eliminated HIV-related restrictions on entry, stay and residence, 48 jurisdictions still maintain various forms of restrictive migration policies for immigrants living with HIV [3]. Some jurisdictions deport non-nationals solely on the basis of their HIV status (Table 1), while others deny both short- and long-term stays on the same grounds (Table 2). Certain jurisdictions may require HIV testing for specific types of permits (Table 3). Unfortunately, when HIV testing is conducted within the context of migration, established global protocols regarding informed consent, confidentiality and counselling are not consistently implemented [7]. This practice overlooks the economic benefits of migration and the sustained productivity of individuals living with HIV, who can benefit from improved treatments during extended stays. Denying entry or deporting someone solely on the basis of their HIV status is considered discriminatory and unjust.

**Table 1:** Countries that deport non-nationals on the basis of HIV

Country	Policy	Outcome (for foreign PLHIVs)
Bahrain	No travel restrictions for tourists; PLHIVs do not need to declare their status to enter the country	- Refusal or revocation of stay permit and deportation
Brunei	- HIV test is required for all work, study and residence permits; positive results will result in deportation	- Refusal or revocation of stay permit and deportation
Cook Islands	- HIV test is required for all work, study and residence permits; positive results will result in deportation	- Refusal or revocation of stay permit and deportation
Egypt	- An HIV test result is required for all work or study visas or for stays > 1 month. Tests performed abroad are usually not recognized	- Refusal or revocation of stay permit and deportation
Iraq	- No HIV test is required for foreigners visiting Iraq for 2 years	- Unclear, likely to lead to deportation
Jordan	- No HIV test is required for short-term stays up to 1 month	.—Denial of residency, deportation. Jordanian citizens who are HIV positive (± their foreign spouses) are supplied with ARVs
Kuwait	- No HIV test is required for short-term stays up to 3 months	- Refusal or withdrawal of residency permit, deportation and permanent barring from re-entry
Malaysia	- Tourists are not required to take an HIV test - For work permits, an initial HIV test is mandatory in the home country and repeated in Malaysia, with annual retesting required	- Denial of permit and possible deportation once HIV is diagnosed in foreigners on the Temporary Employment Visitor Pass
Oman	- Tourists are not required to test for HIV - HIV test results are mandatory for work or residency permits and immigrants who are positive are required to return to their country of origin	- Visitors are allowed to bring their ARVs if positive - Foreign PLHIVs are usually expelled
Qatar	- HIV test is not required for passengers in transit and tourists (< 1 month stay) -Residency and employment applications require HIV test results, and positive results result in denial and deportation	- Denial of residency and employment permits; possible deportation if found to be HIV positive
Russian Federation	- No test required for persons staying for less than 90 days -Stays of more than 90 days (for work or study) as well as multiple entry visas require a negative HIV test result to be approved - Quarterly HIV testing mandated for foreigners residing in the country since 29 December 2021	- Denial of permits and deportation
Saudi Arabia	- Residence and work permit applicants are required to take an HIV test, and permits are not granted if the test returns positive	- Denial of permits and probable deportation
Singapore	- HIV test is not required for tourist or business visa applicants up to 90 days - HIV test is mandatory for all stays beyond 90 days for which a positive test results in rejection	- Entry ban for HIV-positive long-term visitors, possible deportation; HIV-positive foreign spouses are allowed to remain
Solomon Islands	- HIV test is mandatory for visitors staying for more than 90 days	- Possible denial of permit and deportation
Sudan	- No restriction or test requirements for short-term stays (< 3 months) - Applicants of work or residence visas need to submit a negative HIV test result before obtaining such	- Denial of permit and possible deportation
Syrian Arab Republic	-Tourist visa applications typically do not require HIV testing, including 6-month multi-entry visas - Foreigners aged 15–60 must undergo HIV testing when applying for a residence permit, which is granted only if the result is negative -Foreigners intending to marry Syrian nationals must undergo HIV testing	- Denial of residence or long-stay permit, deportation
Turkmenistan	- No restriction or test requirements for short-stay visas, single or multiple entry, up to 12 months - Residency permit for work or study requires an HIV test and a positive HIV test result may lead to deportation	- Denial of permits and possible deportation
United Arab Emirates	- No restriction or test requirements for short-stay travellers, up to 60 days. - -HIV tests are required for foreigners who apply to obtain or renew work and residence permit which is denied, and foreigner is deported once a positive result is returned. Tests performed outside of the UAE are not accepted for this purpose	- PLHIVs are not allowed to enter UAE with ARVs and may be detained and face scrutiny; deportation
Yemen	- Prohibits all HIV-positive persons from entering the nation, irrespective of purpose or duration of stay -No HIV testing requirement for tourists staying less than 3 months - HIV testing mandatory for foreigners applying for residence, work or study permits, including students, employees, refugees and immigrants	- Expulsion from the nation once an HIV test returns positive

Source: Still not welcome – HIV-related travel restrictions Available on https://www.unaids.org/sites/default/files/media_asset/hiv-related-travel-restrictions-explainer_en.pdf

**Table 2:** Countries that may deny both short- and long-term stays on the basis of HIV

Country	Policy	Outcome (for positive PLHIVs)
Aruba	- Short-term visits (30 days) are not subject to an HIV test result - Employment and residency licenses may require an HIV test result for which if returned positive may result in denial of such permits	- Possible denial of work or residence permits
Bosnia & Herzegovina	- Short and/or long-term stay may be prohibited on the basis of a positive HIV status	- Possible denial of work or residence permits
Dominican Republic	- Short-term stay does not require HIV result - Application for work or residence permits require medical checks which include HIV test. A positive HIV test or refusal to get an HIV test usually results in denial of such permits	- Denial of work or residence permits
Indonesia	- HIV test is required for work permits for certain professional groups and are usually denied once result returns positive	- Possible denial of work permits
Kyrgyzstan	- Short-term stay up to 60 days does not require request HIV result - Stay of more than 60 days and all application for work visa must submit an HIV test result. A positive HIV test results in denial of such permits	- Possible denial of work permits
Maldives	- Short-term stay does not require an HIV result - Application for work and residence permits require an HIV test result. A positive HIV test results in denial of residence permit	- Denial of residence permits, Possible denial of work permits
Marshall Islands	- Short-term stay up to 30 days does not require HIV result - All stay for more than 30 days regardless of reason (tourism, work, study, residence) must submit an HIV test result. A positive HIV test results in denial of permit	- Denial of long-term stay permits
Mauritius	- Temporary visitors are not required to submit HIV test result - Application for work and residence permits require an HIV test result. A positive HIV test results in denial of residence permit	- Denial of work and long-term stay permits
St Vincent & the Grenadines	- Short-term visits do not require an HIV test result - Applications for long-term stay require an HIV test result for which if returned positive may result in denial of such permits	- Possible denial of permits for long-term stay
Tunisia	- Short-term visits do not require an HIV test result - Applications for long-term stay (more than 30 days) require an HIV test result for which if returned positive may result in denial of such permits	- Possible denial of permits for long-term stay
Ukraine	- Short-term visits do not require an HIV test result - Anyone with tuberculosis cannot get permanent residency - HIV restrictions on long-term stays are unclear	- Possible denial of permanent residency and other long-term stays for PLHIVs (denial for PLHIVs who have tuberculosis)

Source: Still not welcome – HIV-related travel restrictions Available on https://www.unaids.org/sites/default/files/media_asset/hiv-related-travel-restrictions-explainer_en.pdf

**Table 3:** Countries that may require HIV testing for certain types of permits

Country	Policy	Outcome (for foreign PLHIVs)
Angola	- Short term stays do not require HIV tests - Requirements for a work visa (and likely other long-term stay) includes a medical health certificate including HIV test result.^3^	- Visa for work (and likely other long-term stay) may be denied
Australia	- Students (and their dependents) from sub-Saharan Africa who plan to study for 12 months or more are tested for HIV - People who are 15 years or older must have an HIV test if: • They intend to work or study to train to be doctors, dentists, nurses or paramedics, or • They intend apply for a permanent and provisional visa People who are younger than 15 years, applying for an adoption or other permanent visa must take an HIV test if: • There is a previous history of blood transfusion, • There is any clinical indication of having HIV, or • The biological mother is or was HIV positive Those who have HIV (and/or other conditions such as TB, hepatitis B or C, Hansen’s disease) are required to sign a health undertaking to ensure that they follow-up the health conditions with an onshore health provider, else their application will be rejected	- Possible denial of permits or visa if: • An HIV test is refused, • Treatment for HIV is likely to incur a significant healthcare cost or • The health undertaking is not signed
Azerbaijan	- Short term stays do not require HIV tests - Application for work permit afresh or an extension requires that an employer submits a reference certifying that the foreigner is not infected with HIV, hepatitis B, C - Requirements for a temporary or permanent residence visa includes a reference certifying that the applicant is not a carrier of HIV (hepatitis B and C by extension) and test which must be performed at specified centres in the country	- Denial of extension of work permit as well as residence visa, irrespective of type
Belize	- HIV tests are not required for short-term stays (usually 30 days) - Applications for a permanent residency or citizenship requires submissions of an HIV test result which if positive may result in denial of the application	- Denial of residence permit, some other study or work permits
Cayman Islands	- No requirements for short-term and tourist stays - Applications for long-term stays (more than 3 months) require an HIV test which, if positive, may result in denial of application	- Denial of long-term stay application
Cuba	- No requirements for short-term and tourist stays - Requirements for a long-term stay (more than 3 months) including study visa or an extension of a residency permit requires an HIV test result	- Possible denial of permits for long-term stay, study visa and residency permit
Israel	- Short-term visits do not require an HIV test result - Applications for work permits stay (more than 30 days) require an HIV test result for which if returned positive may result in denial of such permits	- Possible denial of work permits
Kazakhstan	- Visits less than 3 months do not require an HIV test result - Applications for residence permits (and likely work and study permit) require a negative HIV test result to be granted	- Denial of residence permit Possible denial of work and study permit
Lebanon	- Visits less than 3 months do not require an HIV test result - All immigrant labourers are expected to apply for a work permit which must be accompanied by a negative HIV result	- Denial of work permit and possible deportation for immigrant labourers who are PLHIVs
New Zealand	- Visa applicants intending to stay for more than 12 months are required to take an HIV test, but HIV is no longer considered not to meet the immigration acceptable standard of health; hence, visas are unlikely to be denied on the basis of a positive HIV test result. (Effective October 2021)	- Restriction lifted for PLHIVs
Papua New Guinea	- People over 16 years may be required to take an HIV test for entry permit application - An HIV test may be required for a long-term visa (more than 6 months) or for residence permits Entry permit applications may be rejected due to a positive HIV status	- Possible denial of entry permit and long-term visa
Paraguay	- No restriction for short-term tourist stays and no HIV testing on entry - An HIV test is be required for permanent residency applications which will be denied in case of a positive HIV test result	- Denial of permanent residency
Samoa	- An HIV test is required for all forms of stay beyond 90 days • Visitors who indicate that they have tested HIV positive will be subject to questioning by a health professional upon entry	- Possible denial of entry and long-term stays
St Kitts and Nevis	- HIV test is not required for short-term stays - HIV test is required for application for work, study and long-term permits	- Probable denial of work, study or long-term permits
Tonga	- No restrictions for short-term tourist stays - Application for work, study or residence permits require an HIV test result	- Possible denial of entry and long-term stays
Turks and Caicos	- No restrictions for short-term tourist stays (up to 30 days) - Application for stays beyond 30 days usually for residence and/or work permits requires an HIV test - HIV test is required for every renewal of any form of permit	- Denial of work and residency permits, denial of renewal or extension of permits
Tuvalu	- HIV testing is required for work, study and residence permits	- Possible denial of work, study or residence permits

Source: Still not welcome – HIV-related travel restrictions Available on https://www.unaids.org/sites/default/files/media_asset/hiv-related-travel-restrictions-explainer_en.pdf

## Impact of restrictive migration policies on access to HIV prevention, care and treatment services

Immigrating to a foreign country, territory or area presents many challenges. Immigrants often face a multitude of social, financial, legal and political barriers that hinder their access to public services, including healthcare [8]. These healthcare services are essential; however, immigrants living with HIV encounter significant difficulties in accessing treatment, adhering to treatment regimens and benefitting from HIV prevention measures such as regular testing, pre-exposure prophylaxis (PrEP) and post-exposure prophylaxis (PEP) due to restrictive policies in the host country, territory or area [9].

For instance, all long-term visitors to Canada are assessed on the basis of the “excessive demand” they may place on the Canadian healthcare system [10]. A yearly cost exceeding CA$24,057 is deemed “excessive”, yet many publicly supported HIV drugs cost less than this amount [10]. Consequently, a positive HIV test result might make it difficult for a person to permanently immigrate to Canada. Although Canadian laws and policies do not explicitly mention HIV, they do allow for an application to be denied on the basis of medical inadmissibility. Similarly, work permits in St Vincent and the Grenadines are not renewed if an HIV test is positive, and HIV-positive foreigners have no access to treatment or assistance [10].

The lack of access to essential healthcare services is appalling as the immigration process exposes individuals to an increased risk of acquiring HIV infection, compounded by inaccessibility to effective HIV prevention techniques, including early diagnosis through testing, and individuals are less likely to receive treatment [5, 8]. This results in a higher risk of HIV-related mortality among immigrants than a local population [9]. In addition to limited availability of HIV prevention services, other factors heightening the vulnerability of immigrants to HIV infection in host countries can be attributed to various factors, such as social stigma and engagement in risky behaviours [5].

The unavailability of healthcare services for immigrants is further exacerbated by policies that require proof of citizenship or residency [4]. It is unsurprising that undocumented immigrants living with HIV avoid seeking healthcare services for fear of detention, deportation or punishment [4]. In Switzerland, for example, undocumented migrants, like citizens, must obtain expensive private health insurance to access basic healthcare, costing more than €250 a month [4]. Failure to pay premiums on time results in judicial complaints, and obtaining insurance requires verification of identification and residency. When migrants acquire health insurance, their situation may be reported to immigration services.

In the Russian Federation, undocumented migrants have no access to healthcare, which is dependent on the social insurance system [4]. This fear prevents them from seeking HIV testing, treatment and care, significantly increasing the risk of HIV transmission and worsening health outcomes. Additional factors hindering access to HIV prevention and treatment services include provider discrimination, racism, low HIV risk perception, language barriers and negative perceptions [11], which are aggravated by migration policies that discriminate against immigrants living with HIV [12].

Furthermore, policies that demand upfront payment for healthcare services can be a significant barrier to healthcare for immigrants who lack financial means [13] and experience socio-economic disadvantages compared with the general population [14] due to limited access to well-compensated employment opportunities. Lower socio-economic status, whether combined with migration status or not, contributes to health disparities and inferior health outcomes [15]. This situation can impact the ability of immigrants to access HIV prevention, care and treatment services.

Restrictive migration policies can also have an indirect impact on the mental health of immigrants [16], which can subsequently affect HIV prevention and care. This was documented in another study, proving that immigrants are subjected to restrictive integration policies and more prone to negative effects on their mental health, self-rated health, utilization of healthcare services and overall mortality rates [17].

## Recommendations and action

Efforts to improve access to HIV services for immigrants are crucial in achieving Sustainable Development Goal 3, which aims to end the AIDS epidemic by 2030. The World Health Organization (WHO) recognizes that access to the highest attainable standard of health, including HIV prevention, care and treatment services, is a fundamental right of every human being [17]. To achieve this goal, governments of countries with restrictive policies, civil society organizations (CSOs) and international partners should collaborate and take the following actions:

### Government policy evaluation

Governments should conduct a thorough assessment of their migration policies to align with international human rights standards and evidence-based public health measures. This evaluation should ensure that immigrants have equal access to healthcare, remove barriers to HIV prevention, care and treatment services, and address issues related to immigration status, socio-economic status, sexual orientation and gender identity. It is crucial ensure that policies are inclusive and prioritize the consideration of immigrants for employment opportunities, thereby improving their socio-economic status. Specific measures may include removing HIV/AIDS from the list of diseases requiring proof for admission, immigration, employment and reintegration. Governments can also establish discreet health posts that offer specialized care to people living with HIV and those at higher risk of contracting HIV, provide training for healthcare practitioners on migrant health issues, expand outreach programmes targeting vulnerable populations, increase funding for HIV prevention and care activities for immigrants, and facilitate partnerships between governmental and non-governmental organizations to improve service provision for migrant populations.

### Advocacy by CSOs

CSOs should engage in evidence-based advocacy efforts to drive the reform of migration policies that impede the availability of HIV prevention, care and treatment services. These efforts should focus on not only increasing public awareness of the adverse effects of restrictive policies on HIV services but also tackling stigma or any form of discrimination through awareness campaigns and outreach programmes. CSOs should advocate for policy alternatives that promote the rights and wellbeing of immigrants, participate in dialogues with policymakers, and collaborate with relevant stakeholders. Research and data collection should also be prioritized by CSOs to produce robust evidence regarding the impact of migration policies on HIV prevention, care and treatment outcomes.

### Support from international organizations

International organizations should advocate for more inclusive and rights-based migration policies in destination countries, territories or areas. Jurisdictions with restrictive migration policies can benefit from technical support and capacity development provided by international organizations. This may include cultural competence training for healthcare personnel to ensure they understand the unique needs of immigrant populations, establishment of referral networks to facilitate access to comprehensive healthcare services and ensuring that healthcare services are accessible to multilingual and immigrant communities. International organizations should also facilitate collaboration between governments, CSOs and affected communities to address the challenges faced by immigrants in accessing HIV services.

### Research and data collection

Research plays a crucial role in generating evidence to inform policy and practice related to people living with HIV who are migrants. Addressing the significant paucity of data in this area, academic communities must conduct comprehensive research to provide critical insights into health disparities among immigrant populations. Evaluating the effectiveness of various interventions aimed at improving HIV prevention, care and treatment for immigrants is essential. By determining what strategies work best in different contexts, researchers can design and implement more effective health interventions tailored to the unique needs of immigrant communities. Additionally, longitudinal studies tracking health outcomes and the long-term impacts of these migration policies can highlight the benefits of inclusive policies, making a strong case for policy reform.

Collaboration between academia, government bodies, CSOs and affected communities is vital to ensure that research is relevant and impactful. Engaging with communities provides essential perspectives, ensuring studies address real-world challenges and needs. This collaborative approach builds trust and ensures research findings effectively advocate for necessary changes. By addressing these areas, stakeholders can create an inclusive environment where immigrants, especially those living with HIV, have equitable access to essential health services, contributing to the global effort to end the AIDS epidemic by 2030.

## Conclusion

Migration policies affect immigrants’ HIV prevention and care services; hence, a more integrated strategy is required to address these challenges. The exclusion of immigrants from these services hinders worldwide HIV epidemic efforts and promotes HIV transmission among this community. Restrictive and criminalizing laws might discourage immigrants from obtaining assistance. Inclusive migration policies, universal healthcare, outreach activities and a multisectoral approach to HIV prevention and care will enhance immigrants’ and host populations’ health. Countries may enhance immigrants’ health and create a more welcoming atmosphere by following these policy ideas.

## Data Availability

No database or primary data was used in preparing the manuscript.

## Abbreviations

HIV: Human immunodeficiency virus
CSOs: Civil society organizations
UNAIDS: Joint United Nations Programme on HIV/AIDS
AIDS: Acquired immunodeficiency syndrome
PrEP: Pre-exposure prophylaxis
PEP: Post-exposure prophylaxis
PLHIVs: People living with HIV/AIDS
ARVs: Antiretroviral drugs
